# Assessing the impact of COVID-19 on epidemiological changes of severe pediatric respiratory syncytial virus infections in Malaysia

**DOI:** 10.3389/fpubh.2024.1246921

**Published:** 2024-01-31

**Authors:** Chee Mun Chan, Asrul Abdul Wahab, Adli Ali

**Affiliations:** ^1^Department of Pediatrics, Faculty of Medicine, Universiti Kebangsaan Malaysia, Kuala Lumpur, Malaysia; ^2^Research Center, Hospital Tunku Ampuan Besar Tuanku Aishah Rohani, UKM Specialist Children’s Hospital, Kuala Lumpur, Malaysia; ^3^Department of Microbiology, Faculty of Medicine, Universiti Kebangsaan Malaysia, Kuala Lumpur, Malaysia; ^4^Institute of IR4.0, Universiti Kebangsaan Malaysia, Bangi, Malaysia; ^5^Infection and Immunology Health and Advanced Medicine Cluster, Universiti Kebangsaan Malaysia, Kuala Lumpur, Malaysia

**Keywords:** RSV infection, COVID-19, children, immunity debt, preventive restriction

## Abstract

**Introduction:**

Respiratory syncytial virus (RSV) is one of the leading causes of hospitalization and mortality among children with respiratory tract infections. The non-pharmaceutical preventive measures against severe acute respiratory syndrome coronavirus (COVID-19) may have reduced the transmission of RSV, altering its tropical epidemiological seasonality. Thus, this study represents the first attempt to evaluate changes in RSV epidemiology in the context of COVID-19 pandemic in Malaysia.

**Methods:**

Conducted at a tertiary hospital in Kuala Lumpur, Malaysia, this retrospective study analyzed collated data of children aged <12 years who were admitted for severe respiratory infections from 2017 to 2022. Time series models were used to predict the differences between actual and forecasted RSV cases, while logistic regression assessed the statistical association between RSV and COVID-19.

**Results:**

Among the 4,084 children analyzed, we reported a significant inverse relationship between RSV and COVID-19 infections during the pandemic (2020–2021) (*p* < 0.05). In 2020, the RSV positivity rate sharply declined to 8.3 and 5.9%, respectively, in the two prominent seasons. Time series analysis showed a tremendous decrease in cases compared to the expected values, with reductions of 98.3% in the first season and 95.7% in the second season. However, following the lifting of the restriction order in 2022, RSV infections rose sharply with a positivity rate of 36.3%, higher than pre-COVID-19 pandemic levels.

**Conclusion:**

This study provides evidence of increasing RSV cases post-COVID-19 pandemic, due to immunity debt. Hence, the healthcare system must be prepared to address future RSV outbreaks with the appropriate implementation of prophylaxis and public health measures.

## 1 Introduction

Globally, seasonal epidemics of respiratory syncytial virus (RSV) is one of the leading causes of hospitalization and mortality among children, particularly those under the age of 5 (1). The severe manifestations of RSV disease include pneumonia and bronchiolitis, with the latter typically being self-limiting (2). This is supported by the Pediatric Etiology Research for Child Health (PERCH), which identified RSV as the most common pathogen isolated from hospitalized children with severe pneumonia in Africa and Asia, accounting for 31% of all cases (3). In 2015, an estimated 33 million episodes of lower respiratory tract infections (LRTIs) were attributed to RSV infections in children under the age of 5, resulting in 3.2 million hospitalizations and 120, 000 deaths worldwide (4). Despite the known morbidity and mortality associated with RSV, there is currently no approved vaccine to prevent RSV infections (1). Thus, preventive measures aimed at reducing the spread of RSV remain the most promising intervention in controlling these seasonal epidemics.

Respiratory diseases reached a catastrophic milestone with the emergence of severe acute respiratory syndrome coronavirus (COVID-19) in December 2019 in Wuhan, China, impacting millions of adults and children (5). Within 1 year after its emergence, global reported deaths due to this pandemic had reached 5.94 million by 31st December 2021. Interestingly, this figure was hypothesized to be underestimated by a factor of 3.07 based on excess mortality rates, which is the net difference between the actual number of deaths during the pandemic and the expected number based on past trends in all-cause mortality (6). In Malaysia, COVID-19 was first detected in January 2020 and surged in March 2020, prompting the government to implement Movement Control Order (MCO) to restrict mass movements and gatherings in combating the pandemic.

Importantly, COVID-19 shares a similar air-borne transmission mechanism with RSV (1). Therefore, the preventive recommendations implemented during the MCO to mitigate COVID-19 transmission may have also helped to preventing local transmission of RSV. Notably, the United States reported historically low weekly percentages of positive RSV rates early in the pandemic (<1.0% per week compared to approximately 12–16% during the pre-COVID era) while an Australian study also observed decreased RSV activity due to COVID-19 restrictions (7, 8). Regionally in Asia, China also experienced two sharp declines in RSV infections during the two national outbreaks of the COVID-19 pandemic (9). In our previous epidemiological study, we observed a sharp decline in RSV cases in 2020, possibly due to reduced exposure to RSV as a result of the nationwide lockdown. (10).

The COVID-19 pandemic has presented a unique opportunity for the widespread implementation of public health interventions on a global scale for a limited period of time (1). However, there is a lack of evidence regarding the effectiveness of these public health measures in local and regional contexts, contributing to the decreased transmission of RSV during the pandemic. As a result, the widened epidemiology gap has intrigued researchers worldwide to comprehend the possible shift in RSV seasonality in assessing the timing and effectiveness of prophylactic and therapeutic interventions. To address this knowledge gap, it is crucial to establish large-scale surveillance that can improve our understanding of RSV epidemiology, particularly in the context of COVID-19. Therefore, our study represents a pioneering effort aimed at advancing the comprehension of disease epidemiology within the framework of the COVID-19 pandemic, particularly regarding RSV. We aim to potentially identify a larger scale or a shifted paradigm of RSV infections compared to previous years in Malaysia.

## 2 Materials and methods

### 2.1 Study design and sample size

This was a retrospective study conducted at Hospital Canselor Tuanku Muhriz (HCTM), a tertiary hospital located in the Klang Valley spanning three states namely Selangor, Kuala Lumpur and the Federal Territorial State of Kuala Lumpur (11). Based on the latest hospital audit report (2018–2021), we received an average of 518, 885.8 patients per year, with 14.9% (77, 300.8 patients) being pediatric patients aged 0 to 17 years old (12). For this study, we analyzed data collected over a six-year period, from 1st January 2017 to 31st December 2022. We included 4,096 hospitalized patients ranging from birth up to 12 years old who underwent nasopharyngeal analysis (NPA) due to acute respiratory tract infections (ARTIs). Additional demographic information, including date of birth, race, test date, and age at the time of the test, was extracted from patient databases. Children with mild respiratory symptoms and not requiring non-invasive ventilation (NIV) or without NPA analysis were excluded from this study. We further excluded 12 repeated RSV samples obtained from the same patient within a two-week timeframe, hypothesized to be from the same period of infection. Consequently, only the earlier RSV samples were included for analysis. Using the formula for population sampling by Krejcie and Morgan, a 95% confidence interval from a final sampling frame of 4,084 patients required at least 354 positive samples. This study received ethical approval and support from the Secretariat of Research and Innovation Universiti Kebangsaan Malaysia (UKM) (Project code: JEP-2021-780).

### 2.2 RSV infection detection

ARTI is defined as the presence of cough and cold with respiratory symptoms such as rapid breathing, tachypnoea above age limit, and/ or chest in-drawing, along with warning signs such as the inability to tolerate feeding, persistent vomiting, lethargy, or stridor (10). Symptomatic children with moderate to severe respiratory distress symptoms requiring NIV were admitted, and NPA was routinely obtained to detect various common respiratory viruses. We utilized the direct fluorescent antibody (DFA) method, specifically the D3 Ultra DFA Respiratory Virus Screening and Identification Kits (Diagnostic Hybrids, United States) to detect RSV, adenovirus, influenza A and B, and parainfluenza 1, 2, and 3 viruses (sensitivity: 95.5%, specificity: 98.3%) (13).

### 2.3 COVID-19 epidemiological data

The daily COVID-19 cases spanning from 1st March 2020 to 31st December 2022 were extracted from the Department of Statistics, Ministry of Health Malaysia (14). To depict the pinnacle of COVID-19 infections, our dataset exclusively encompassed the daily count of newly confirmed positive cases across all age groups, ranging from pediatric to adult populations. Moreover, our analysis refrained from stratifying the data according to state-specific case aggregates, despite the study being conducted in Kuala Lumpur.

### 2.4 Public health measures in response to COVID-19 pandemic

**Figure 1:**
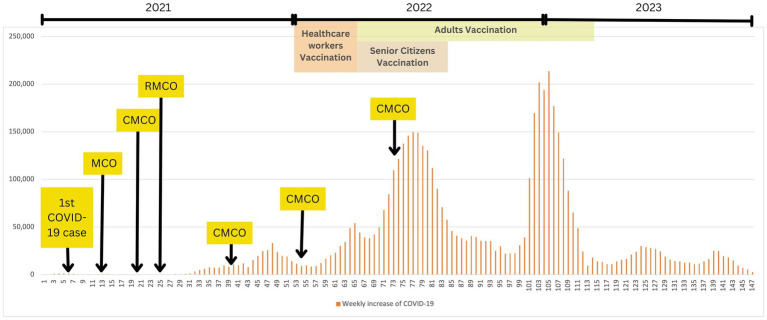
COVID-19 and public health measures timeline in Malaysia (1st March 2020 – 31st December 2022). This graph illustrates the weekly increase of COVID-19 cases within the stipulated timeframe, where number of weeks, *N* = 147. Data was obtained from the Department of Statistics, Ministry of Health Malaysia. MCO, movement control order; CMCO, controlled movement control order; RMCO, recovery movement control order.

On 4th February 2020, Malaysia recorded its first locally transmitted case of COVID-19, which subsequently increased sharply to a consistent daily count surpassing 100 in March 2020. As a result, the Malaysian government strictly implemented a nationwide movement control order (MCO) on 18th March 2020 to mitigate the spread of the virus. This measure included restrictions on mass movements and gatherings across all locations. Despite the initial measures, virus transmission persisted, leading to more stringent enforcement on 1st April 2020. As a result of improved compliance with the MCO, a notable decrease in daily new COVID-19 cases and an increase in recoveries were observed 14 days after its enforcement. To revive the national economy while continuing to manage the situation, more businesses were allowed to resume operations, leading to the revision as conditional MCO (CMCO) which was further relaxed to recovery MCO (RMCO) (15). Interestingly, COVID-19 detection rates increased exponentially from late 2021 to early 2022 due to an increased number of clusters, including prison inmates, foreigners, and mass gatherings related to elections. In response, the government proactively mapped and detect active cases, leading to several mass sampling areas nationwide. The Malaysian nationals also implemented the use of online contact tracing, “MySejahtera,” to assist in COVID-19 outbreak (Figure 1) (16).

### 2.5 Statistical analysis

We performed the data analysis using the Statistical Package for the Social Sciences (SPSS) version 26 (IBM, Chicago, IL, United States). Descriptive analyses were utilized to present the demographic data, where categorical variables were expressed as frequencies and percentages, and continuous data as means. We stratified our RSV data into three cohorts: pre-COVID (2017–2019), during-COVID (2020 and 2021) and post-COVID (2022). To visualize the trends and relationships within each cohort, we plotted the number of RSV and COVID-19 cases on time-series graphs, using a weekly timeframe. Consequently, we calculated the positivity rate of RSV during two prominent seasons using the following equation:


$$\left(\mathrm{Total}\;\mathrm{positive}\;\mathrm{cases}/\mathrm{Total}\;NPA\;\mathrm{samples}\;\mathrm{collected}\right)\times 100\%$$

In addition, we performed a time series analysis to forecast the total counts of RSV cases for the years 2020, 2021, and 2022. This analysis was based on the weekly counts of RSV cases from 2017 to 2019, focusing on the two seasons of interest. To generate the predictions, we estimated the model parameters using the maximum likelihood method and 95% confidence intervals (CIs) to account for the uncertainty in the forecasted values. This was done by calculating the percentage difference between the two numbers, employing the following equation:


$$\left(\mathrm{Observed}\;\mathrm{frequency}/\mathrm{Expected}\;\mathrm{frequency}\right)\times 100\%$$

In order to establish the statistical association between COVID-19 and RSV cases, we conducted a Pearson correlation analysis. Subsequently, significant correlations were further examined using bivariate logistic regression analysis. To determine the significance of the correlations, a two-sided value of p of less than 0.05 was considered statistically significant.

## 3 Results

### 3.1 Demographic data of severe respiratory syncytial virus infections for the past 6-year period

Among these 4, 084 samples, 697 (17.1%) children tested positive for RSV. Demographically, the median age of positive cases was 1 year, with the majority of cases being under 2 years old. The highest RSV detection rate was observed among children under 6 months (23.9%). Analysis of the data showed a positive trend of increasing RSV cases from 2017 to 2022, except for a slight dip in 2020 (15.0%). This decline may be attributed to underreporting or possibly the impact of COVID-19 public health measures. Overall, Malay children exhibited a higher rate of RSV infection (17.7%). A summary of the demographic data for RSV-positive cases can be found in Table 1.

**Table 1:** Demographic information of children with severe RSV infections.

Indicator (*N* = 4,084)	RSV positive = 697, *n* (%)
*Age*	
<6 months	172 (23.9)
6 months to 2 years	397 (20.1)
2.1 years to 5 years	95 (8.9)
>6 years	33 (10.2)
*Year*	
2017	47 (8.9)
2018	56 (10.8)
2019	167 (17.7)
2020	80 (15.0)
2021	85 (20.2)
2022	262 (22.9)
*Ethnicity*	
Malay	653 (17.7)
Chinese	27 (13.9)
Indian	4 (8.2)
Other	13 (8.8)

Descriptive analysis was used to describe the demographic information of RSV-positive children based on (i) age groups (ii) year of analysis, and (iii) ethnicity. The results were expressed in *n* (%), where *n* represents the number of children.

### 3.2 Prominent seasonality of respiratory syncytial virus infections before COVID-19

**Figure 2:**
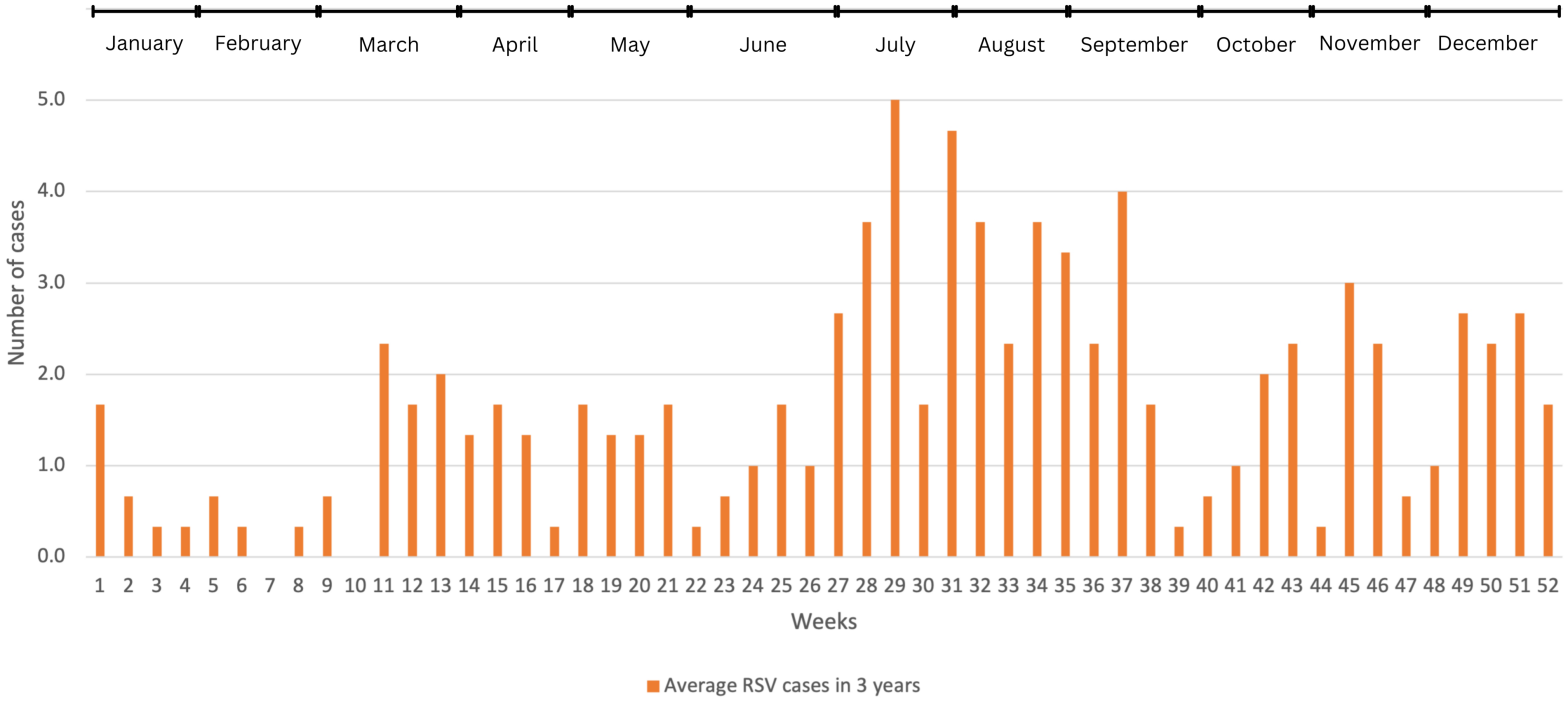
The average number of RSV cases before COVID-19 in weekly basis (2017–2019). We used descriptive analysis to compare the average number of RSV-positive cases within 52 weeks in these 3 years. The results were expressed as (*n*), where *n* represents the average number of cases.

Visually, Figure 2 shows that RSV infections occurred consistently throughout the year, exhibiting two distinct periods of pronounced seasonality. The first period of seasonality spanned from week 26 to week 31, occurring in the middle of the year. The second period of seasonality was observed from week 45 to week 52, toward the year-end. Using this pattern as a baseline for comparison, the data from the years during-COVID (2020, 2021) and post-COVID (2022) were analyzed to illustrate the trend of the infectivity rate during these two seasons.

### 3.3 The relationship between RSV and COVID-19 infections In subsequent 3 years (2020–2022)

**Figure 3:**
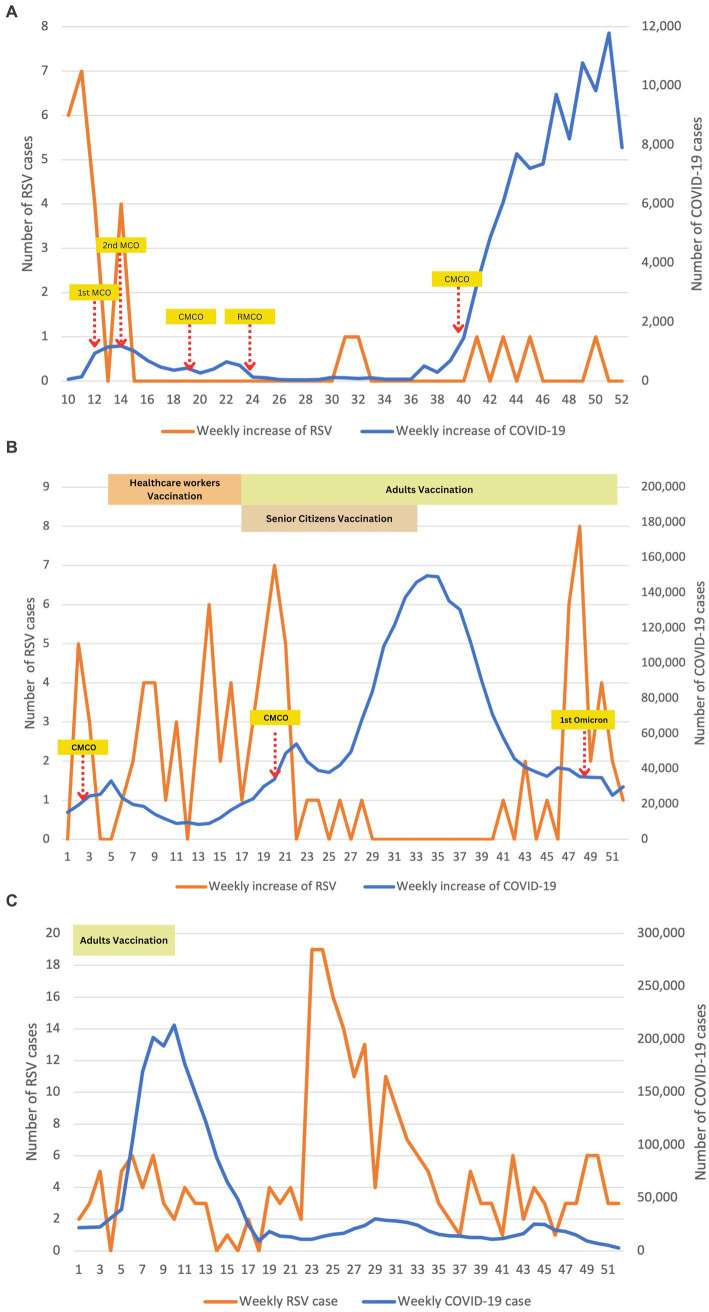
Epidemiological time-series plot of COVID-19 and RSV infections during COVID-19 (2020–2022). **(A)** Epidemiological data of COVID-19 and RSV in 2020. **(B)** Epidemiological data of COVID-19 and RSV in 2021. **(C)** Epidemiological data of COVID-19 and RSV in 2022. We performed descriptive analysis to demonstrate the weekly positive cases of RSV and COVID-19 during study periods from 2020 to 2022. Data were plotted on time-series graphs to depict the relationship between the two viruses. Months (weeks) description: January (1–5); February (6–9); March (10–14); April (15–18); May (19–22); June (23–27); July (28–31); August (32–35); September (36–40); October (41–44); November (45–48); December (49–52). MCO, movement control order; CMCO, controlled movement control order; RMCO, recovery movement control order.

Figure 3 illustrates an inverse relationship between COVID-19 and RSV infections. This relationship is particularly significant in Figure 3A (2020) and Figure 3B (2021), where RSV infections drastically declined as COVID-19 infections increased (*p* < 0.001). In 2020, the average number of RSV infections dropped significantly to approximately 1 case throughout the year. Overall, the highest RSV infection among our patients was observed at the beginning of the COVID-19 outbreak, coinciding with the implementation of lockdown measures. As strict lockdowns were enforced during the 2nd MCO, the number of COVID-19 cases gradually decreased and plateaued to less than 100 new cases daily from week 24 to 36. This led to a gradual easing of restrictions, transitioning to the CMCO and subsequently to the RMCO. Around 4 weeks after the RMCO, RSV infections resurfaced after remaining dormant since March 2020. However, the overall number of recorded RSV cases during this period was relatively lower than pre-COVID. Statistically, logistic regression supported this trend and predicted that with every increase of COVID-19 cases in 2020, we noted a significant drop in the number of RSV cases, approximately by 8-fold (*p* < 0.014).

Large-scale immunizations against COVID-19 were enforced nationwide in the first quarter of 2021 to all adults aged 18 and above, leading to herd immunity against COVID-19. Consequently, the infection rate of COVID-19 significantly reduced to approximately 10, 000 cases per week. However, this decrease in COVID-19 cases indirectly contributed to a surge in RSV infections among our patients. However, this phenomenon was only observed until the second half of 2021 when a sudden spike in COVID-19 cases necessitated the reimplementation of MCO. Interestingly, RSV rebounded to 8 cases at week 46, marking the highest count since the COVID-19 pandemic started. However, it declined sharply in the following week, potentially attributed to the detection of the Omicron variant of the virus in Malaysia. The presence of the Omicron variant may have played a role in the subsequent decrease in RSV cases until early 2022.

As the vaccination coverage among the adult population increased and the number of COVID-19 cases decreased, the MCO was fully lifted in the second half of 2022. As anticipated, there was a sharp rebound in the total number of RSV cases, with more than 10 cases per week reported in week 23. This high number of RSV cases persisted consistently for approximately 2 months, as depicted in Figure 3C.

### 3.4 The impact of COVID-19 on trend of RSV infections in two prominent seasonalities

To assess the impact of COVID-19 on RSV infections, we stratified the overall cases based on the two seasons of RSV epidemics. In pre-COVID era (2017–2019), RSV exhibited its highest positivity rate during the first season, with a rate of 20.6%. However, with the emergence of COVID-19, we observed a significant decline in the percentage positivity of RSV during the same season, dropping to 8.3% (Table 2). This decline is further supported by our time series model, indicating that the number of RSV cases in 2020 was 98.3% lower than the predicted value. A similar pattern was observed in the second season of 2020, resulting in 95.7% fewer cases than predicted.

**Table 2:** Comparison of percentage positivity and time-series analysis for prediction of RSV cases in 3 years (2020–2022).

Year	Positivity rate^a^ (%)	Actual number of cases	Predicted number of cases	Percentage difference^b^ (%)	Upper limit (95% CI)	Lower limit (95% CI)	*R* ^2^
*Week 26–31*							
2017–2019	20.6						
2020	8.3	1	58	98.3	270	−153	0.715
2021	18.2	2	78	97.4	306	−149	
2022	36.3	62	98	63.3	341	−144	
*Week 45–52*							
2017–2019	14.8						
2020	5.9	2	47	95.7	165	−70	0.839
2021	22.2	24	63	61.9	189	−64	
2022	14.4	28	78	64.1	213	−57	

^a^Percentage positivity was calculated based on the total number of positive RSV cases and the total number of NPA samples collected. The rate was expressed in percentage (%). ^b^Percentage difference was derived from analysis of two time series models between actual and predicted number of RSV cases in two seasons. The percentage difference was expressed in (%).

As the restriction orders were gradually lifted in 2021, we observed a gradual increase in the trend of RSV infections. Towards the end of the year, total RSV infections peaked and surpassed the pre-COVID era, reaching a positivity rate of 22.2%. This realignment with the postulated trend resulted in the actual cases differed by 61.9% of the expected value, as shown in Table 2.

In 2022, when no movement restrictions were implemented, we observed the re-emergence of RSV with the recurrence of seasonality. Notably, the overall positivity rate of RSV during the first season of 2022 was significantly higher than the pre-COVID era, reaching a rate of 36.3%.

## 4 Discussion

Severe RSV infections have indirectly posed a substantial economic burden on healthcare systems, governments. and society (17). Many studies have shown that the disease burden extends beyond affected children, impacting caregivers, leading to a loss of work productivity and increased hospitalization costs (17). Butel et al. estimated an average total cost per patient of around EUR 2000 (equivalent to USD 2163) for the first episode of acute bronchiolitis, mainly attributed to hospitalization costs (18). Prior to COVID-19, RSV accounted for 5.4% of all detected positive respiratory pathogens in the United States between December 2019 and March 2020. However, during the implementation of public health measures to combat COVID-19, the RSV positivity rate dropped dramatically to 0.03% from December 2020 to March 2021 (19). Besides, studies in Spain and Germany reported that preventive measures implemented against COVID-19 resulted in fewer hospitalizations for RSV bronchiolitis during the autumn-winter season of 2020 to 2021 (20, 21). In Asia, Japanese investigators reported a significant reduction in RSV infections among children aged between 0 to 11 months, which had highest prevalence before the pandemic. South Korean researchers reported an 81 and 91% reduction in RSV-positive cases during 2020 (22, 23). Therefore, these findings align with a growing body of evidence suggesting that stringent public health measures can effectively reduce the spread of epidemic respiratory viruses (24).

In our latest epidemiological study, we identified two peaks of RSV seasonality occurring in distinct monsoon periods, specifically during July to August and October to December, coinciding with previous local and regional studies. (10, 25–27). Surprisingly, in this study, we observed an unusual increase of RSV cases throughout the first half of 2021. This is deemed as a global phenomenon as many countries reported a change in the seasonal variation of RSV during the COVID-19 pandemic (28). Typically, RSV infections peak during colder temperatures and reduced humidity, conditions favorable for the stability and transmission of the virus (10). However, in Shanghai, China, Ran Jia et al. documented an unusual increase in the RSV detection rate during the summer of 2021 (28). Interestingly, in Japan, there was a shift in RSV cases occurring in the spring of 2021, with a higher magnitude compared to the pre-COVID-19 period (29). Similarly, in Taiwan, Lee et al. observed a delay in the RSV season, with cases occurring during the winter of 2020–2021 instead of the usual peaks in spring and fall (30).

Several factors have been identified as responsible for the seasonality change in RSV and its unusual resurgence. Firstly, the relaxation of public health measures has revealed a strong association with increased RSV activity (31). The return of children to schools and the lifting of social gathering restrictions indirectly contribute to the transmission of RSV among children. Moreover, many studies have emphasized the role of adults as reservoirs for RSV, which was previously underestimated (31). During the COVID-19 period, public health measures were strictly implemented on older children and adults for better compliance than younger children (32). Consequently, while adults benefited from the easing of restrictions earlier in 2021, RSV cases increased significantly among younger children who remained restricted due to closed childcare facilities. This raises speculation that adults play a major role in household chains of RSV transmission.

Secondly, the substantial decrease in protective immunity, termed as immunity debt, resulted from extended periods of low exposure to pathogens (33). The children’s immune systems have now weakened due to reduced exposure to pathogens, a consequence of the public health response to the pandemic (34, 35). This immunity debt poses a particular concern for RSV in younger children, especially with the waning of maternal antibodies and a lack of seasonal exposure, rendering them susceptible to future and potentially more severe infections (35). In addition to public health measures, the phenomenon of viral interference may also help to explain the sudden disappearance of RSV in the context of COVID-19 (36). Briefly, it has long been hypothesized that respiratory viral infections can prevent superinfection of other respiratory pathogens through to the activation of innate immunity, mainly via interferon response. This is widely evidenced by the delay of Influenza Virus (H1N1) in 2009 during the first pandemic by Rhinovirus in September–October 2009 period (37). Accordingly, we agree with the hypothesis proposed by Raffaella et al. that the sharp decline in RSV circulation may have been partly contributed by the ongoing spread of the highly contagious and abundant COVID-19 Omicron variant surge in late 2021, particularly affecting unvaccinated children (36).

Although the implementation of public health measures has disrupted the transmission of RSV and COVID-19, it is unlikely that these measures can entirely eliminate the infections (31). One classic example to emulate is the Ebola Virus disease (EVD), where the African health authorities, despite being highly prepared to manage the endemic after several regional outbreaks over the last decade, still maintain extreme vigilance to avoid cross-border exportation of EVD and further international lockdowns triggered by COVID-19 (38). Moreover, it is crucial to highlight the importance of protecting immunocompromised children who are at a higher risk of RSV and other infections, while also being cautious of the ongoing prevalence and high contagiousness of COVID-19 within our communities (34, 39–42). To date, Palivizumab, a recombinant humanized monoclonal antibody targeting RSV F-protein, is the only passive immunization that could reduce the rate and severity of RSV infections when administered intramuscularly to children as pre-exposure prophylaxis (31). Over the years, Palivizumab has been clinically recommended for high-risk children under 2 years of age, including preterm infants and infants with congenital heart disease and chronic lung disease. Considering the shift in RSV seasonality following the emergence of COVID-19, the American Academy of Pediatrics (AAP) recommended more than five consecutive doses of Palivizumab for the best efficacy (43). Hence, it is imperative for clinicians to determine the optimal timing of RSV immunization, in light of the changed seasonality, as a promising pharmaceutical strategy for preventing RSV infections.

We have identified several limitations in our study. Firstly, due to the retrospective nature of the study spanning a period of 6 years, we were unable to ensure consistent nasal swab testing for all children with symptoms of ARTI. This may have resulted in underestimating the true burden of RSV infection, particularly during the COVID-19 pandemic period. Secondly, our reported data on COVID-19 cases were based on daily national statistics instead of state-focused data, which may limit the accuracy of assessing the relationship between COVID-19 and RSV cases in the exact locality. Nevertheless, it is worth noting that our study area, which covered Kuala Lumpur and Selangor, remained one of the highest contributors to daily COVID-19 cases throughout the three-year study period (44). Regretfully, we did not exclusively demonstrate pediatric COVID-19 infection as Malaysia was lagging in COVID-19 detection rate compared to other developed countries, thus the main catchment area was to aim at adults, rather than pediatric population. In addition, our study focused solely on RSV infection, and we were unable to comprehensively assess the epidemiological characteristics of other common respiratory viruses before and during the pandemic. This includes the detection of any co-infections by our DFA kit, which could have influenced the clinical outcomes of the patients. Although our diagnostic RSV test used has commendable sensitivity (95.5%) and specificity (98.3%), false negatives and false positives of the results may affect the actual prevalence from what we reported. During the pandemic, COVID-19 was detected using real-time polymerase chain reaction (PCR), which have higher sensitivities than DFA detection of RSV, thus explained the large ratio between COVID-19 and RSV positive cases. The impact of these inaccuracies can vary depending on the context in which the test is used, the prevalence of RSV in the population, and the potential consequences of misdiagnosis.

## Data availability statement

The original contributions presented in the study are included in the article/supplementary material, further inquiries can be directed to the corresponding author.

## Ethics statement

This study received ethical approval and support from the Secretariat of Research and Innovation Universiti Kebangsaan Malaysia (UKM) (Project code: JEP-2021-780).

Written informed consent for participation was not required for this study in accordance with the national legislation and the institutional requirements.

## Author contributions

AA: Conceptualization, Funding acquisition, Project administration, Supervision and Writing- review & editing; CM: Data curation, Formal analysis, Investigation, Methodology, Writing-original draft; AW: Conceptualization, Supervision, Writing- review & editing. All authors contributed to the manuscript revision, read, and approved the submitted version.
